# Occurrence of Typical Domestic Animal Viruses in Wild Carnivorans: An Emerging Threat to the Conservation of Endangered Species

**DOI:** 10.1155/2024/3931047

**Published:** 2024-02-07

**Authors:** Nathana B. Martins, Julio C. Neves de Almeida, Marianne S. S. Gonçalves, Lana I. Gila, Débora R. Yogui, Mario H. Alves, Arnaud L. J. Desbiez, Paulo E. Brandão, Aline S. da Hora

**Affiliations:** ^1^Laboratory of Veterinary Etiological Investigation, School of Veterinary Medicine, Federal University of Uberlandia, Uberlandia 38408-100, Brazil; ^2^Instituto de Conservação de Animais Silvestres (ICAS), Mato Grosso do Sul 79070-180, Campo Grande, Brazil; ^3^Postgraduate Program in Ecology and Conservation, Federal University of Mato Grosso do Sul, Mato Grosso do Sul 79070-900, Campo Grande, Brazil; ^4^Nashville Zoo, Nashville 37211, TN, USA; ^5^Royal Zoological Society of Scotland (RZSS), Murrayfield, Edinburgh EH12 6TS, UK; ^6^Department of Preventive Veterinary Medicine and Animal Health, School of Veterinary Medicine, University of São Paulo, São Paulo 05339-003, Brazil

## Abstract

Wild species are susceptible to several typical domestic animal pathogens, and the increasingly close contact between these groups is a predictive factor for disease exposure. Some viruses are important and old-known, and others are emerging or reemerging for domestic carnivorans and have been identified as threats to the conservation of wild mammals. The purpose of the study was to investigate the occurrence of bocaparvoviruses (BoVs, *Parvoviridae* family, *Parvovirinae* subfamily, *Bocaparvovirus* genus), parvoviruses (*Parvoviridae* family, *Parvovirinae* subfamily, *Protoparvovirus* genus, *Protoparvovirus carnivoran1*), hepadnaviruses (*Hepadnaviridae* family), coronaviruses (*Coronaviridae* family, *Orthocoronavirinae* subfamily), paramyxoviruses (*Paramyxoviridae* family) and canine distemper virus (*Orthoparamyxovirinae* subfamily, *Morbillivirus* genus, *Morbillivirus canis*), poxviruses (*Poxviridae* family), feline herpesvirus (*Orthoherpesviridae* family, *Alphaherpesvirinae* subfamily, *Varicellovirus* genus, *Varicellovirus felidalpha1*), feline calicivirus (*Caliciviridae* family, *Vesivirus* genus, *FCV*), feline immunodeficiency virus (*Retroviridae* family, *Orthoretrovirinae* subfamily, *Lentivirus* genus, *FIV*), feline leukemia virus (*Retroviridae* family, *Orthoretrovirinae* subfamily, *Gammaretrovirus* genus, *FeLV*), and gammaherpesviruses (*Orthoherpesviridae* family, *Gammaherpesvirinae* subfamily) in wild carnivorans. A total of 30 biological samples from the families Canidae, Felidae, Mephitidae, Mustelidae, and Procyonidae were evaluated. All animals were victims of vehicular collisions in the state of Mato Grosso do Sul, Brazil. Canine parvovirus (CPV-2) DNA was detected in the spleen of a bush dog (*Speothos venaticus*), a jaguarundi (*Puma yagouaroundi*), and a jaguar (*Panthera onca*), FeLV proviral DNA was found in the spleen of an ocelot (*Leopardus pardalis*); while CDV RNA was detected in the liver of a jaguarundi. Phylogenetic analysis carried out with the partial sequence of the CPV-2 VP2 gene and the U3 (LTR) gag region of FeLV showed 100% identity with strains obtained from domestic dogs and cats, respectively. The approximation between wild and domestic animals favors the transmission of pathogens, especially between phylogenetically close species, such as members of the Canidae and Felidae families. Identification of the DNA and RNA of potentially fatal viruses such as CPV-2, FeLV, and CDV in four wilds endangered to extinction and understudied species contributes to our understanding of the pathogens circulating in this free-ranging and vulnerable population.

## 1. Introduction

Infectious diseases represent an emerging threat to the conservation of wild mammals [1]. Several pathogens typical in domestic animals can impact wild species and the increasingly frequent contact between these groups may facilitate the exchange of pathogens among evolutionarily related hosts, such as members of the Canidae and Felidae families [2]: bocaparvoviruses, parvoviruses, hepadnaviruses, coronaviruses (CoVs), paramyxoviruses, canine distemper virus (CDV), poxviruses, feline herpesvirus (FHV-1), feline calicivirus (FCV), feline immunodeficiency virus (FIV), feline leukemia virus (FeLV), and gammaherpesviruses are old and significant viral pathogens, emerging and reemerging, of extreme clinical relevance for domestic carnivorans [3–7].

Bocaparvoviruses and parvoviruses belong to the *Parvoviridae* family, and both are important and resilient viral pathogens of domestic and wild animals [8]. Canine parvovirus (CPV-2, *Protoparvovirus carnivoran1*) is highly pathogenic and often fatal, associated with gastroenteritis. It can also lead to myocarditis due to viral infection of the dividing myocardial cells, resulting in sudden death or congestive heart failure [9]. Additionally, cerebellar hypoplasia has been rarely reported in dogs after in utero infection, but is more common in kittens infected with feline parvovirus (FPV) [10]. The *Protoparvovirus carnivoran1* species encompasses various viruses, including CPV-2, FPV, mink enteritis virus (MEV), and raccoon parvovirus (RPV). These viruses pose a significant threat to endangered wild carnivorans, including Siberian tigers [8]. The bocaparvoviruses are capable of infecting various hosts, resulting in a spectrum of clinical manifestations that range from asymptomatic infections to severe illness that may progress to fatality [7, 11–15].

Hepadnaviruses comprise a group of ancient DNA viruses of the *Hepadnaviridae* family that infect diverse taxonomic groups, including humans (*hepatitis B virus* (*HBV*)), with significant implications for public health due to its associations with chronic liver diseases and hepatocellular carcinoma [16]. Novel hepadnaviruses have been identified in domestic cats and dogs, indicating their circulation among domestic carnivorans, although their presence in wild populations remains undocumented [6, 17, 18].

CoVs (*Coronaviridae* family, *Orthocoronavirinae* subfamily) comprise the largest single-stranded RNA viruses infecting mammals [19]. Over the last two decades, three newly identified CoVs have sparked human epidemics characterized by substantial morbidity and mortality. These viruses are believed to have originated from animals, particularly bats [20, 21]. The potential for cross-species transmission and public health threats posed by CoVs underscore the importance of vigilant monitoring, especially considering the incomplete understanding of CoVs specific to wild animals [22–26].

Paramyxoviruses, a group of emerging single-stranded RNA viruses within the *Paramyxoviridae* family, hold global importance for human and animal health. The identification of diverse and novel paramyxoviruses circulating among wildlife reservoir species has been on the rise. These viruses occasionally cross over into other terrestrial mammals, including humans [27]. Among the paramyxoviruses affecting animals, CDV (*Morbillivirus canis*) poses a threat to wild carnivorans populations of conservation concern. This includes documented instances of CDV affecting lions (*Panthera leo*) [28, 29], African wild dogs (*Lycaon pictus*) [30–32], and black-footed ferrets (*Mustela nigripes*) [33].

The poxviruses (*Poxviridae* family) represent one of the largest DNA viruses capable of infecting a broad spectrum of hosts. Typically, these infections lead to cutaneous lesions, skin nodules, or a disseminated rash, with the potential for fatal outcomes [34]. Various species, including carnivorans and especially those in the Felidae family, are susceptible to poxvirus infections [35–38]. The recent *Monkeypox virus* outbreak highlights the global threat of poxviruses [39], with novel members identified in new hosts, such as Brazilian porcupines infected with the *Brazilian porcupinepox virus (Oryzopoxvirus* genus), underscoring the severity of poxvirus infections [40].

Herpesviruses constitute an emerging virus within the order *Herpesvirales* [41]. Feline herpesvirus (*Orthoherpesviridae* family, *Alphaherpesvirinae* subfamily, *Varicellovirus* genus, *Varicellovirus felidalpha1*) is a significant contributor to feline upper respiratory tract disease (FURTD), affecting both domestic and wild felids across various geographical regions [42, 43]. Wild brazilian felids have also been associated with feline herpesvirus infections [44]. Gammaherpesviruses (*Orthoherpesviridae* family, *Gammaherpesvirinae* subfamily) have a preference for targeting host lymphocyte cell populations, and their infection may result in morbidity or mortality, with a high prevalence observed in wild felids [45, 46].

FCV (*Caliciviridae* family, *Vesivirus* genus, *FCV*) has a single-stranded RNA genome and plays a crucial role in FURTD, contributing to stomatitis and oral ulcers. Cats infected with highly virulent strains of FCV can also develop FCV-associated virulent systemic and fatal disease [47]. Several species of domestic and wild felids have been exposed to FCV, with a notably high prevalence observed in some endangered species, including free-ranging lions (*P. leo*) and Iberian lynx (*Lynx pardinus*) [42, 43, 48–50].

FIV (*Orthoretrovirinae* subfamily, *Lentivirus* genus) and FeLV (*Orthoretrovirinae* subfamily, *Gammaretrovirus* genus) are important retroviruses capable of cross-species transmission among both domestic and wild felid populations [50–53]. They elicit a spectrum of illnesses, including immune system suppression, lymphoma and leukemia development, and increased mortality [54, 55]. FIV and FeLV pose potential threats to vulnerable species, exemplified by their impact on the Iberian lynx (*L. pardinus*) [48], while FeLV is recognized as a threat to the conservation of the Florida panther (*Puma concolor coryi*) [56, 57]. Documented exposure to retroviruses exists in various felid populations. Both FIV and FeLV were detected in the guigna (*Leopardus guigna*) in Chile [58], and FIV was identified in pumas (*P. concolor*) in Brazil [44].

Our hypothesis is that medically relevant viruses are circulating in the vulnerable population of free-ranging carnivorans in the Brazilian Cerrado and Pantanal biomes. It is worth noting that some hosts sampled for this study are poorly studied or have not been studied at all due to the difficulty in capturing these animals because of their cryptic behavior and/or the limited number of individuals *in situ*. Therefore, this study aims to investigate the occurrence and genetic diversity of the viruses bocaparvoviruses, parvoviruses, hepadnaviruses, CoVs, paramyxoviruses, CDV, poxviruses, feline herpesvirus, FCV, FIV, FeLV, and gammaherpesviruses in carnivorans found as roadkill in Mato Grosso do Sul, Brazil.

## 2. Materials and Methods

### 2.1. Sampling Area and Animals

This study was carried out with samples provided by Instituto de Conservação de Animais Silvestres (ICAS), all from wild animals killed by cars on highways in the state of Mato Grosso do Sul, Brazil, in the period 2018–2023.

Biological samples (spleen, liver, lung, nervous tissue (brain), skin, and feces) from 30 individuals from the families Canidae—crab-eating fox (*Cerdocyon thous* (*n* = 5)), maned wolf (*Chrysocyon brachyurus* (*n* = 1)), bush dog (*Speothos venaticus* (*n* = 1)), and hoary fox (*Lycalopex vetulus* (*n* = 1)); Felidae—Pampas cat (*Leopardus colocolo* (*n* = 1)), ocelot (*Leopardus pardalis* (*n* = 2)), jaguar (*Panthera onca* (*n* = 2)), and jaguarundi (*Puma yagouaroundi* (*n* = 4)); Mephitidae—Molina's hog-nosed skunk (*Conepatus chinga* (*n* = 1)); Mustelidae—neotropical otter (*Lontra longicaudis* (*n* = 4)), tayra (*Eira barbara* (*n* = 1)); and Procyonidae—South American coati (*Nasua nasua* (*n* = 5)), and crab-eating Raccoon (*Procyon cancrivorus* (*n* = 2)) were aseptically collected with sterile material and stored in nuclease-free microtubes. The approximate duration between sample collection and storage was around 2 hr.

The universal sample for all pathogens was the spleen, as it was available from all individuals in the study. In some cases, additional samples, including the liver, lung, central nervous system, and feces, were also tested. Skin samples from selected individuals were tested for poxviruses due to the tropism of this pathogen for cutaneous tissue.

All samples were stored at −20°C and then sent to the Laboratory of Veterinary Etiological Investigation of the School of Veterinary Medicine of the Federal University of Uberlândia in a thermal box with dry ice. The biological materials were kept at −80°C until processing.

### 2.2. Processing of Samples and Extraction of Genetic Material

Organ samples were macerated, diluted in a suspension with nuclease-free water, in the proportion of 70% organ and 30% water, and subsequently subjected to three cycles of freezing in liquid nitrogen and heating in a thermoblock at 55°C before extraction. The stool samples were processed into a suspension with nuclease-free water, subjected to three cycles of refrigeration, and rested for 5 min at room temperature. Then, the organ and fecal samples were clarified by centrifugation at 12,000 x *g* for 16 min at 4°C. A total of 200 *µ*L supernatant was collected and used for the extraction of total DNA and RNA using the PureLink™ Mini Kit Viral RNA/DNA (Invitrogen™, Thermofisher), according to the manufacturer's protocol.

### 2.3. Molecular Assays

Endogenous control for PCR and RT-PCR reactions was carried out by amplification of a fragment of the GAPDH (glyceraldehyde-3-phosphate dehydrogenase) gene [59] and of the *β*-actin mRNA [60], respectively.

Reverse transcriptase followed by real-time PCR (RT-qPCR) for detection of CDV [61], feline coronavirus (FCoV) [62], FIV [63], FeLV [64, 65], and FCV [66] were performed using GoTaq® 1-Step RT-qPCR System (Promega Corporation), according to the manufacturer's protocol. Real-time PCR (qPCR) reactions to detect DNA of provirus FIV and FeLV, CBoV [67], parvoviruses [68–70], feline herpesvirus [71] and gammaherpesviruses [72], hepadnaviruses [73], and poxviruses [74] were performed using the GoTaq® qPCR Master Mix (Promega Corporation), according to the manufacturer's protocol.

The Pan-paramyxoviruses [75] and Pan-CoV [62] RT-PCR assays were performed using SuperScript™ III Reverse Transcriptase (Invitrogen™, ThermoFisher), according to the manufacturer's protocol. Subsequently, a Nested-PCR was performed using GoTaq® Green Master Mix (Promega Corporation), as per the manufacturer's protocol.

The specific feline viruses (FIV, FeLV, FCV, FCoV, and feline herpesvirus) were tested exclusively in felid samples.

As a negative control, nuclease-free water was used. Commercial vaccines for dogs (Vanguard®, Zoetis) and cats (Feline-4, Boehringer Ingelheim) were used as positive controls for Pan-paramyxoviruses, CDV, parvoviruses, CoV, and feline herpesvirus. As positive controls for FIV and FeLV, we utilized samples known to be routinely positive in the laboratory. Additionally, CBoV (ON571563) and Brazilian porcupinepox virus (MN692191) positive samples were included. An HBV-positive sample, generously supplied by Dr. João Renato Rebello Pinho (Israelita Albert Einstein Hospital), was used as the hepadnaviruses control. The gammaherpesviruses positive control was kindly provided by Prof. Dr. João Pessoa Araújo Junior (Instituto de Biociências, Universidade Estadual Paulista Júlio De Mesquita Filho).

The positive sample for FeLV was subjected to a DNAse treatment (RQ1 RNase-Free DNase, Promega) according to the manufacturer's instructions, followed by RNA extraction using TRI Reagent BD (Sigma–Aldrich), as per the manufacturer's protocol. Then, it was subjected to RT-qPCR for the detection of FeLV RNA. Positive samples for CDV, CPV-2, and FeLV were submitted to PCRs targeting the region of the gene that encodes the N protein [61], the VP2 protein [69, 70], and U3 (LTR) gag gene [65], respectively, using GoTaq® Green PCR Master Mix (Promega Corporation) to produce the amplicons to be sequenced.

### 2.4. Sanger Sequencing

Amplicons from CPV-2 and FeLV were excised from the agarose gels and purified using the PureLink™ PCR Purification Kit (ThermoFisher Scientific). For CDV, purification was conducted using ExoProStar (Cytiva™ Life Sciences), according to the manufacturer's instructions. The DNA sequencing reaction was performed bi-directionally on a capillary sequencer (Genetic Analyzer 3500, Applied Biosystems) using the manufacturer's instructions for a commercial kit (BigDye™ Terminator v3.1 Cycle Sequencing Kit, ThermoFisher Scientific). Chromatograms were generated from each of the sequences in duplicate and evaluated and edited using FinchTV 1.4.0 (Geospiza, Inc.; Seattle, WA, USA; http://www.geospiza.com) and BioEdit v.7.2.5 [76], only Phred ≥20 fragments were used. The chromatograms were manually analyzed and edited, and the final sequences of each sample were obtained with the Cap-contig application of BioEdit.

### 2.5. Phylogenetic Analysis

The search for homology between the sequence of this study and other sequences deposited in GenBank was carried out using the BLAST® 2.13.0 tool. Nucleotide and amino acid alignments of the CPV-2 VP2 and the FeLV U3 (LTR) gag were performed using the Clustal W application of BioEdit. The phylogenetic tree was constructed using MEGA software v.11.0.10 [77]. The set of aligned nucleotide sequences was submitted to the Find Best-Fit Substitution Model in MEGA to determine the best evolutionary model.

For CPV-2 and FeLV, the maximum likelihood method was used. For CPV, the Tamura 3 model was applied, and for FeLV, the Kimura 2 model was utilized. For both, 1,000 bootstrap repetitions were conducted.

## 3. Results

A jaguarundi (*P. yagouaroundi*) spleen sample (RK1415) was excluded from the study because of the absence of amplification in the *β*-actin mRNA control, and a spleen sample from another jaguarundi showed a weak band in the same assay, despite this, was included in the study.

**Figure 1:**
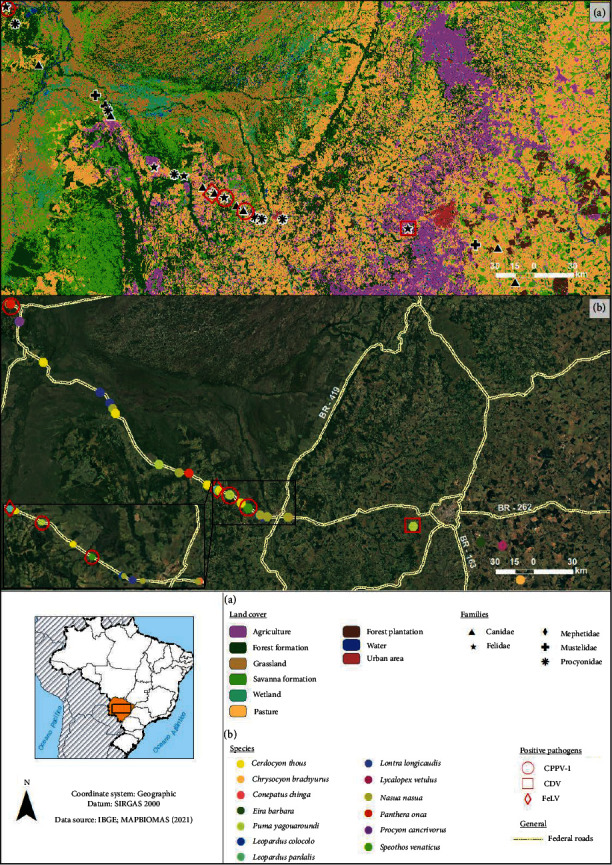
Location and landscape of free-ranging wild carnivorans species sampled on highways in the state of Mato Grosso do Sul, Brazil, from 2017 to 2021. The viruses found in the respective hosts are displayed.

CPV-2 was detected in three spleen samples (3/29; 10.34%) from a jaguarundi, a bush dog (*S. venaticus*), and a jaguar (*P. onca*) with a quantification cycle (Cq) of 18, 10, and 15, respectively. A spleen sample (1/29; 3.45%) from an ocelot (*L. pardalis*) tested positive for FeLV proviral DNA and negative for RNA. The integrity of the sample was confirmed through the endogenous control test, utilizing RT-PCR for *β*-actin mRNA. Additionally, a liver sample of jaguarundi (1/29; 3.45%) was positive by RT-qPCR for CDV, with a Cq value of 40 (Table 1). The spatial locations of the taxonomic groups sampled and the pathogens found in the present study are shown in Figure 1.

Due to the insufficient amount of jaguarundi sample, it was possible to perform CPV-2 sequencing only on the bush dog sample. In the RT-qPCR analysis for CDV, we encountered a high Cq (40 cycles) and observed low fluorescence (0.89 dF/dT). These outcomes are likely due to the limited amount of viral RNA present, which is a consequence of the sampling method and RNA degradation post-mortem. Regrettably, these conditions have impeded our efforts to successfully amplify this sample in CDV sequencing reactions.

**Figure 2:**
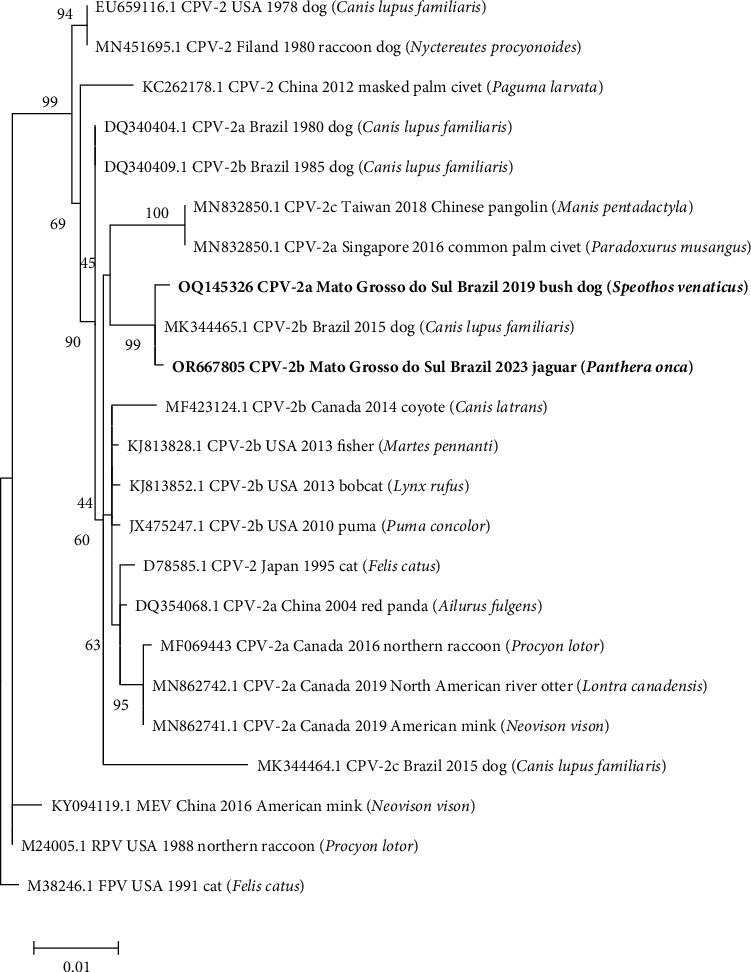
Phylogenetic tree based on 23 partial nucleotide sequences (1,203 nts) of the gene encoding the CPV-2 VP2 protein, constructed with the maximum likelihood method through the Tamura 3 model [35] in MEGA v. software 11.0.10 [33]. The numbers next to each node represent the values of 1,000 bootstrap repetitions [36]. The scale represents the number of substitutions per site. The samples are identified with the GenBank accession number, viral variant, geographic region, year, and host species. The sequences of this study are identified in bold.

The nucleotide sequence (1,548 nts and 1,395 nts) of CPV-2 obtained from bush dog (OQ145326) and jaguar (OR667805) showed 99.8% and 99.93% identity with CPV-2b (MK344465.1) and 99.55% and 99.86% with CPV-2a (MF177241.1), respectively. Notably, both strains are from domestic dogs in the South of Brazil, indicating that the variants detected in the wildlife species of the present study (bush dog and jaguar) share a high identity with strains circulating in domestic dogs from a distinct region in the country. According to the analysis of amino acid residues, the sequence from bush dog was classified as the CPV-2a variant, while the sequence from jaguar was classified as the CPV-2b variant (Table 2); however, according to a new proposed classification [78], both strains can also be classified in clade W.4 (Figure 2).

**Figure 3:**
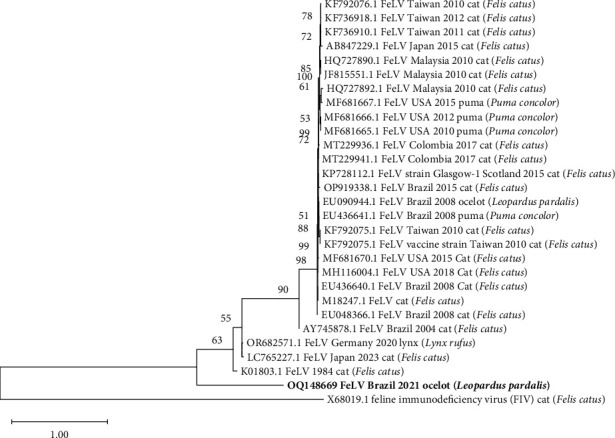
Phylogenetic tree based on 14 partial nucleotide sequences (489 nts) of the U3 (LTR) gag region of FeLV, constructed with the maximum likelihood method using the Kimura 2 model software MEGA v. 11.0.10 [33]. The numbers next to each node represent the values of 1,000 bootstrap repetitions [36], with only those greater than 50% shown. The scale represents the number of substitutions per site. Samples are identified with GenBank accession number host species and geographic region. The sequence of this study is identified in bold.

The FeLV nucleotide sequence (569 nts) of the U3 (LTR)-gag gene obtained from ocelot (OQ148669) showed 98.95% identity with a domestic cat from Colombia (MT229941.1) (Figure 3).

## 4. Discussion

The present study demonstrates the detection of genetic material from CPV-2 in jaguarundi (*P. yagouaroundi*), bush dog (*S. venaticus*), and jaguar (*P. onca*); the FeLV provirus in ocelot (*L. pardalis*); and RNA of the CDV in jaguarundi *P. yagouaroundi* in Mato Grosso do Sul, Brazil.

Mato Grosso do Sul in Brazil is a tropical climate region with high seasonal variation, including three main biomes: Pantanal in the northwest, Cerrado in the center, and Atlantic Forest in the east and southeast [79, 80]. The state boasts a diverse array of mammals, including 166 species distributed across 31 families [81]. However, little is known about the viral pathogens that circulate in free-living mammals in the region. Additionally, the literature is limited to serological studies and the detection of morbilliviruses in *L. vetulus* and *C. thous* [82, 83]. Other viruses had not been previously investigated in free-living carnivorans in the study area.

In other free-living carnivorans, such as the coati (*N. nasua*), river otter (*L. longicaudis*), crab-eating fox (*C. thous*), and even in captive jaguarundi (*P. yagouaroundi*), molecular detection of CPV-2 has been described. It ranges from asymptomatic infection to a diversity of clinical presentations, including gastroenteric clinical signs, culminating in mortality [44, 84–87]. These same species were sampled in this study but with the occurrence of protoparvoviruses restricted only to jaguarundi, possibly reflecting the number of animals tested for each species. Despite this, this finding demonstrates the circulation of this ubiquitous virus with high environmental resistance in the studied region. Although CPV-2 is an old acquaintance in small animal veterinary medicine, the feline variant (FPV) has recently been reclassified as a reemerging pathogen [88], causing a series of outbreaks with high mortality in different countries around the world after several decades of silence.

The FeLV U3 (LTR) gag region of the *L. pardalis* sequence (OQ148669) exhibited a higher nucleotide identity (98.95%) with a sequence obtained from a domestic cat in Colombia (MT229941.1). Studies have previously demonstrated the presence of FeLV proviral DNA in captive ocelot (*L. pardalis*) in southern Brazil, in the free-living jaguar (*P. onca*) in a different region of the Pantanal of Brazil, in captive oncilla (*Leopardus tigrinus*) and jaguarundi, associated with pulmonary, hepatic, renal and splenic alterations [89–91]. FeLV infection in the present study was classified as regressive due to the presence of proviral DNA and the absence of RNA [92].

CDV RNA has been detected in hoary fox (*L. vetulus*) and *C. thous* run over in the same study area, exhibiting neurological, gastroenteric, and respiratory clinical presentation [82, 83] and in a maned wolf (*C. brachyurus*) with clinical gastroenteric and respiratory signs [93]. In South America, CDV has already been detected in fecal samples of jaguarundi (1/5; 20%) and ocelot (3/71; 4%), both free-living [93], indicating circulation in free-ranging felids in different regions of South America.

Although not detected, CBoV, CoV, FIV, feline herpesvirus, gammaherpesviruses, hepadnaviruses, and poxviruses represent emerging or reemerging and relevant viruses that can cause important infections in domestic and wild animals. Therefore, there is a growing need for surveillance studies of these pathogens, given the significant gap in the literature on infectious diseases in free-ranging wild animals [40, 94–98].

The main impacts of CPV-2, FeLV, and CDV on wild species include high mortality, immunosuppression, decreased fertility due to early mortality of young individuals, and reduced population turnover. These threats are particularly significant for populations of species already threatened with extinction, such as bush dog (*S. venaticus*), jaguarundi (*P. yagouaroundi*), jaguar (*P. onca*), and ocelot (*L. pardalis*). Futhermore, these species are rarely encountered in the wild, and capturing *L. pardalis* is challenging, hindering research on several individuals from wild populations. Consequently, this limitation restricts the development of comprehensive and specific conservation strategies [5, 57, 99–102].

These species are classified into different degrees of threat, a global perspective by the IUCN Red List, where only bush dog (*S. venacitus*) and jaguar (*P. onca*) were classified as Near Threatened with a declining population. While these global assessments provide an overview, it is crucial to consider regional and national evaluations. In Brazil, IBAMA (Brazilian Institute of Environment and Renewable Natural Resources) classified three of the positively identified species—jaguar, bush dog, and jaguarundi—as vulnerable [103]. However, the status of the ocelot (*L. pardalis*) remains the least concern, although regional lists identified it as a declining and vulnerable population outside the Amazon biome [103, 104].

The ecological characteristics of the landscape directly influence the dynamics of pathogens and hosts, with urban areas demonstrating a higher density of susceptible hosts (dogs and cats), thus favoring the occurrence of viruses primarily transmitted through direct contact and at high population densities [102]. Contrastingly, rural areas usually have a lower abundance of wild animals and may include domestic animals that can act as sources of both food and infectious agents [93, 102].

It is crucial to note that domestic carnivorans are recognized as the primary reservoirs of CPV-2, FeLV, and CDV [105–107]. Notably, all wild animals examined in this study were found in significantly altered environments, encompassing both urban and rural settings. These environments displayed a scarcity of native vegetation, underscoring the extensive fragmentation and human impact on the landscape (Figure 1). Wild carnivorans positive for FeLV and protoparvovirus were discovered in areas close to pastures, often victims of road accidents. In contrast, a jaguarundi positive for CDV was found in an agricultural area. This study serves as a poignant example that the coexistence of wild and domestic carnivorans in the same area can heighten the risk of pathogen transmission, leading to the spread and persistence of certain domestic animal pathogens in free-ranging mammals [82, 108].

Nonetheless, wild hosts within the order Carnivora were also identified as free-living reservoirs, playing a crucial role in sustaining the pathogen in the environment [109]. This dynamic elevates the risk of pathogen transmission between species that share the same geographical area. The wild carnivorans in the current study were discovered in close proximity to one another, indicating the potential for the exchange of pathogens among individuals circulating in these shared areas.

A high degree of amino acid identity (100%) was observed with the CPV-2 reference variants, especially in molecular markers corresponding to amino acid residues (aa) 87, 93, 101, 267, 300, 305, and 440. S297N aa substitution in VP2 also observed in CPV-2 lineages in South America, plays a key role in host–pathogen interactions, especially in immune response evasion mechanisms [110–112]. The Y324L substitution previously reported in CPV-2b from dogs in southern Brazil [110] is situated in a region proximal to the binding site of the canine transferrin receptor (TfR). This region is subject to positive selective pressure and has the capacity to directly impact the interaction of the virion with the TfR, thereby influencing the range of hosts [113, 114]. The amino acid residue 426 has been identified as a major mutation site for CPV-2 evolution, where antigenic differences are observed between CPV-2a, CPV-2b, and CPV-2c variants: CPV-2 and CPV-2a have asparagine (Asn), CPV-2b has aspartate (Asp), and CPV-2c has glutamate (Glu) [115, 116]. In the present study, asparagine was observed at this residue in the variant obtained from the bush dog, and aspartate in the variant from the jaguar, classifying them as CPV-2a and CPV-2b, respectively.

The risks of pathogen transmission from domestic animals to wildlife can be mitigated by implementing measures such as vaccination against viruses that are significant to domestic animals [117]. Although all the viruses observed in this study (CPV-2, FeLV, and CDV) are preventable through vaccination, a substantial portion of the dog and cat population in Brazil consists of unvaccinated animals.

## 5. Conclusions

Genetic material from CPV-2, FeLV, and CDV has not previously been detected in free-living *P. yagouaroundi*, *S. venaticus*, and *L. pardalis* in Brazil. These species are relatively understudied in terms of viral pathogens and are classified to some degree as threatened with extinction.

Studies investigating viral pathogens in free-living wild mammal populations must be conducted, incorporating molecular and phylogenetic characterization. This approach is essential to comprehend the variants circulating in wild hosts, facilitating the application of more effective measures to mitigate or contain the circulation of these viruses. To this end, utilizing samples obtained from animals that are victims of vehicular collisions is both feasible and advisable.

## Figures and Tables

**Table 1:** Species of wild free-ranging carnivorans sampled (*n* = 30) in the present study regarding collection date, gender, estimated age group, sample types, and results of molecular assays.

ID	Collection date	Species	Sex	Age	Samples	PCR/RT-PCR
NEC 76	2018	Molina's hog-nosed skunk (*Conepatus chinga*)	F	Adult	Spleen	—
NEC 79	2018	Jaguarundi (*Puma yagouaroundi*)	M	Adult	Liver	CDV +
NEC 98	2019	Maned wolf (*Chrysocyon brachyurus*)	M	Young	Spleen	—
NEC 104	2019	Jaguarundi (*P. yagouaroundi*)	M	Adult	Spleen	CPV-2 +
NEC 105	2019	Bush dog (*Speothos venaticus*)	M	Adult	Spleen	CPV-2 +
NEC 106	2019	Hoary fox (*Lycalopex vetulus*)	NI	Young	Liver	—
NEC 112	2019	South American coati (*Nasua nasua*)	F	Young	Spleen	—
NEC 114	2019	Neotropical otter (*Lontra longicaudis*)	F	Adult	Spleen	—
NEC 118	2019	Crab-eating raccoon (*Procyon cancrivorus*)	F	Adult	Spleen	—
NEC 119	2020	Neotropical otter (*L. longicaudis*)	F	Adult	Spleen	—
NEC 120	2020	Tayra (*Eira barbara*)	F	Adult	Spleen	—
NEC 125	2021	Ocelot (*Leopardus pardalis*)	M	Adult	Spleen	FeLV +
NEC 127	2021	Jaguarundi (*P. yagouaroundi*)	M	Adult	Sp, Sk	—
NEC 129	2021	Neotropical otter (*L. longicaudis*)	F	Adult	Sp, Sk, Br, Lu, F	—
NEC 130	2021	Pampas cat (*Leopardus colocolo*)	F	Adult	Spleen, skin	—
NEC 131	2021	South American coati (*Nasua nasua*)	M	Adult	Spleen	—
NEC 132	2021	Crab-eating raccoon (*P. cancrivorus*)	M	Adult	Spleen	—
NEC 133	2021	Ocelot (*L. pardalis*)	M	Adult	Feces and skin	—
NEC 139	2021	Neotropical otter (*L. longicaudis*)	M	Adult	Spleen	—
RK1371	2021	Crab-eating fox (*Cerdocyon thous*)	M	Adult	Spleen	—
RK1401	2021	Crab-eating fox (*C. thous*)	M	Adult	Spleen	—
RK1403	2021	Crab-eating fox (*C. thous*)	M	Adult	Spleen	—
RK1405	2021	South American coati (*N. nasua*)	F	Adult	Spleen	—
RK1391	2021	Crab-eating fox (*C. thous*)	M	Adult	Spleen	—
RK1415	2021	Jaguarundi (*P. yagouaroundi*)	M	Adult	Spleen	Exclude *β*-actin mRNA control
RK1435	2021	South American coati (*N. nasua*)	F	Adult	Spleen	—
IHP 1	2022	Jaguar (*Panthera onca*)	M	Adult	Spleen	—
IHP 2	2023	Jaguar (*P. onca*)	M	Adult	Spleen	CPV-2 +
CDMF	2023	Crab-eating fox (*C. thous*)	M	Adult	Spleen	—
QUATIF	2023	South American coati (*N. nasua*)	F	Young	Spleen	—

*Note*: ID: identification; MS: Mato Grosso do Sul state; F: female; M: male; NI: not identified; Br: brain; Sp: spleen, Sk: skin, Li: liver, Lu: lung, F: feces; -: negative. The underlined letters indicate the exclusion of the sample by *β*-actin mRNA control.

**Table 2:** Amino acid residues in the CPV-2 VP2 capsid protein used to characterize and identification of viral variants.

Aminoacid residues											
Variant/GenBank accession number/host	87	93	101	267	297	300	305	324	375	426	440
**FPV** (M38246.1) cat (*Felis catus*)	M	K	I	***F***	S	A	D	Y	***D***	N	***T***
**MEV** (KY094119.1) vison (*Neovison vison*)	M	K	***T***	***F***	S	V	D	Y	***D***	N	R
**RPV** (M24005.1) raccoon (*Procyon lotor*)	M	K	***T***	***F***	S	A	D	Y	***D***	N	T
**CPV-2** (M38245.1) dog (*Canis lupus familiaris*)	M	***N***	I	***F***	S	A	D	Y	N	N	N
**CPV-2a** (MT981029.1) dog (*C. l. familiaris*)	***L***	***N***	***T***	***F***	A	***G***	***Y***	***L***	***D***	N	***T***
**CPV-2b** (MT981028.1) dog (*C. l. familiaris*)	***L***	***N***	***T***	***F***	A	***G***	***Y***	Y	***D***	D	***T***
**CPV-2c** (MF177250.1) dog (*C. l. familiaris*)	***L***	***N***	***T***	***F***	A	***G***	***Y***	Y	***D***	E	***T***
**CPV-2a (OQ145326) bush dog (*Speothos venaticus*)**	***L***	***N***	***T***	***F***	***N***	***G***	***Y***	***L***	***D***	N	***T***
**CPV-2b (OR667805) jaguar (*Panthera onca*)**	***L***	***N***	***T***	***F***	***N***	***G***	***Y***	***L***	***D***	D	***T***
**CPV-2a** (MF177241.1) dog (*C. l. familiaris*)	***L***	***N***	***T***	***F***	***N***	***G***	***Y***	Y	***D***	N	***T***
**CPV-2b** (MK344465.1) dog (*C. l. familiaris*)	***L***	***N***	***T***	***F***	***N***	***G***	***Y***	***L***	***D***	D	***T***
**CPV-like** (KJ813892.1) coyote (*Canis latrans*)	***L***	***N***	***T***	***F***	A	***G***	***Y***	Y	***D***	D	***T***
**CPV-like** (KJ813881.1) gray wolf (*Canis lupus*)	***L***	***N***	***T***	***F***	A	***G***	***Y***	Y	***D***	D	***T***
**CPV-like** (KJ813890.1) red fox (*Vulpes vulpes*)	***L***	***N***	***T***	***F***	A	D	D	Y	***D***	N	***T***
**FPV** (KJ813869.1) puma (*Puma concolor*)	***L***	***N***	***T***	***F***	A	***G***	***Y***	Y	***D***	E	***T***
**FPV** (AJ249557.1) cheetah (*Acinonyx jubatus*)	M	K	***T***	***F***	S	A	D	Y	***D***	N	***T***
**FPV** (OM810196.1) tiger (*Panthera tigris*)	M	K	***T***	***F***	S	A	D	Y	***D***	N	***T***
**FPV** (KP019620.1) small Indian civet (*Viverricula indica*)	M	***N***	***T***	***F***	S	A	D	Y	***D***	N	***T***
**FPV** (MN862749.1) North American river otter (*Lontra canadensis*)	M	K	***T***	***F***	S	A	D	Y	***D***	N	***T***
**CPV-like** (KJ813836.1) fisher (*Martes pennanti*)	***L***	***N***	***T***	***F***	S	D	D	Y	***D***	N	***T***
**New CPV-2b** (JX411926.1) beech marten (*Martes foina*)	***L***	***N***	***T***	***F***	A	***G***	***Y***	Y	***D***	D	***T***
**RPV** (KM083041.1) raccoon dog (*Nyctereutes procyonoides*)	M	***N***	I	***F***	S	A	D	Y	***D***	N	***T***

*Note*: A, alanine; C, cysteine; D, aspartic acid/aspartate; E, glutamic acid/glutamate; F, phenylalanine; G, glycine; H, histidine; I, isoleucine; K, lysine; L, leucine; M, methionine; N, asparagine; P, proline; Q, glutamine; R, arginine; S, serine; T, threonine; V, valine; W, tryptophan; Y, tyrosine. The bold italicized cells in the table represent the amino acid identity with the CPV-2 strain from bush dog (*Speothos venaticus*) and jaguar (*Panthera onca*) obtained in the present study.

## Data Availability

The sequences for CPV-2 of *S. venaticus* and *P. onca* and FeLV of *L. pardalis* are available at GenBank as OQ145326, OR667805, and OQ148669, respectively.
